# Being a Postgraduate Nursing Student in Limpopo Province, South Africa: An Exploratory Study

**DOI:** 10.3390/nursrep14030121

**Published:** 2024-06-30

**Authors:** Tshepo Albert Ntho, Charity Ngoatle, Tebogo Maria Mothiba, Lina Sebolaisi Hlahla, Thabo Arthur Phukubye, Mamare Adelaide Bopape

**Affiliations:** 1 Department of Nursing Science, University of Limpopo, Sovenga 0727, South Africa; 2 Faculty of Health Sciences, University of Limpopo, Sovenga 0727, South Africa

**Keywords:** postgraduate nursing students, experiences, open and distance learning, journey

## Abstract

Research proficiencies for nurses include the ability to search for and evaluate evidence, disseminate findings, and apply findings to practice within the context of caring. **Aim**: This study explored the experiences of distant postgraduate nursing students in Limpopo Province, South Africa. **Methods**: The population consists of all students registered for postgraduation studies in a nursing programme at two South African universities in Limpopo Province. Purposive sampling was used to select 23 registered postgraduate nursing students. Data were collected through unstructured one-on-one interviews, including an audio recorder and field notes. The narrative data from interviews were analysed qualitatively through Tesch’s open coding descriptive qualitative data analysis method. **Results**: Three main themes emerged: the reasons behind engaging in postgraduate studies, factors impacting postgraduate studies’ success, and a description of challenges associated with conducting research. **Conclusions**: The students generally have varied experiences about whether or not completing a research project is a valuable learning experience. The relationship between a postgraduate student and their research supervisor is crucial to ensuring that the students advance consistently and complete their theses on time. The study will, therefore, assist HEIs offering postgraduate programmes in nursing in realising the challenges faced by students and coming up with strategies to combat the challenges.

## 1. Introduction

Globally, there has been a focus on the value of conducting research in Higher Education Institutions (HEIs). Notably, research is an essential and effective method for finding solutions to socio-economic issues faced by communities, including understanding and improving the current practice in the health fraternity [1,2]. In addition, nurses should consider the Sustainable Development Goals (SDGs), particularly SDG 4, which aims to ensure inclusive and high-quality education for all people and to promote lifelong learning [3]. According to Mbombi and Mothiba, many academically orientated nurses aspire to achieve postgraduate qualifications, which include master’s and doctoral degrees [4]. Postgraduate qualifications allow nurses to advance to more senior positions, improve working conditions, elevate their occupational prestige, and increase the likelihood of better salaries [5]. 

Sikongo, Ashipala, and Pretorius state that postgraduate enrolment in nursing has advantages for students and organisations and influences patient care [6]. Therefore, postgraduate nursing research curricula have an essential role in advancing nursing science through research initiatives and implementing Evidence-Based Practice (EBP) to improve healthcare services and patient care [7]. Evidence-based practice, or EBP, is a method of providing healthcare in which healthcare providers make clinical decisions for particular patients based on the best available evidence or the most pertinent information [8]. EBP entails making thoughtful, nuanced decisions based on patient preferences, circumstances, and characteristics in addition to the body of available evidence. However, few nurses aspire to enrol in postgraduate research education (Master’s and PhD). For example, in the American Mobile Nurses (AMN) survey in 2019, it was revealed that only 18% of the 20,000 nurses who took part in the study were enrolled in a postgraduate education program, and only one in five of them had plans to continue their education to become an advanced nurse practitioner [9]. Similarly, in South Africa, a qualitative study conducted in a selected HEI indicated that registered nurses lacked information concerning postgraduate nursing qualifications and did not see the purpose of obtaining a master’s or doctoral degree [4]. 

The Open Distance Learning (ODL) mode in the nursing fraternity and other professions is essential for addressing the demands of employees who have a high need for education but cannot complete it through the formal or regular educational system [9]. In most cases, these employees include low-income students who want to advance their education, who want to learn and earn at the same time, and who live far from HEIs and are not limited to employees who are parents but still want to advance their education and obtain degrees [10]. Therefore, it is for this reason that HEIs are committed to adopting ODL as the primary mode of widening participation and addressing broader educational problems in general, as contended by Lephalala and Makoe, including Mwale-Mkandawire [11,12]. Interestingly, the ODL philosophy is a flexible educational system that could increase opportunities for the unreached student population by allowing them to study and conceptualise their research in more convenient locations and times [13]. 

Although the ODL mode is perceived as an effective mode for higher education, it is associated with challenges. These challenges include high student dropout rates, lack of access to research materials, lack of immediate feedback, and lack of research mentoring [14]. Hence, Armstrong-Mensah, Ramsey-White, Yankey, and Self-Brown are adamant that making distance learning work for all students is challenging, as postgraduate students are disconnected from institutional services [15]. As a result, postgraduate students may find it difficult to conceptualise and understand the research process, which is not limited to developing academic writing skills since the Master’s and PhD programmes are offered on full research basis. Although ODL has advantages in the postgraduate education system, some HEIs have been experiencing various challenges. In India, the challenges of ODL faced by students have been documented in the study conducted by Roy, Ray, and Rabidas, which include challenges relating to administrative activities, such as lack of interaction with a mentor, delayed feedback, and others [16]. 

Similarly, the study by Ilonga, Ashipala, and Tomas about challenges experienced by students studying through ODL at an HEI in Namibia also revealed numerous difficulties for students who study in ODL mode [14]. These challenges go beyond information technology-related issues, such as slow internet connectivity, and are not limited to mentor time-related and HEI-related challenges. Nevertheless, ODL remains a fundamental mode of study promoting access to postgraduate education in HEIs worldwide. 

There is a dire need for more postgraduate research throughput and completion in South Africa [17]. According to Strebel and Shefer, South Africa continues to perform poorly compared to other countries regarding postgraduate research throughput, timely completions, and reduction of dropout rates among postgraduate students [18]. Previous researchers such as Bopape, Khauoe, and Obaje and Jeawon have cited many factors that affect and hinder the postgraduate research throughput and completion rate at South African HEIs [19,20,21]. Nursing is no exception due to the poor completion rate and throughput among postgraduate students. According to Mbombi and Mothiba, the nursing profession is still experiencing challenges concerning postgraduate research enrolment, completion, and throughput rate [4]. 

ODL has grown exponentially over the past few years, even though it raises significant challenges in the technological research arena [10,14]. In the Limpopo province, HEIs are categorised as “historically disadvantaged” and face several challenges concerning postgraduate research throughput, completion, and dropout rate [22]. For instance, Singh is adamant that rural universities lack research capacity building, and efforts to improve the postgraduate research throughput and timely completion are complex [23]. These difficulties include insufficient research mentoring, underqualified research supervisors, and financing. Therefore, the current paper sought to explore and describe the experiences of postgraduate nursing students in conducting research as an ODL journey at previously disadvantaged HEIs of the Limpopo province. 

## 2. Materials and Methods 

A qualitative research approach with an explorative, descriptive design was used to understand the phenomenon under study. The exploratory–descriptive research design enabled the researcher to understand the experiences of postgraduate nursing students in conducting research as an ODL journey at two HEIs in the Limpopo province of South Africa [24]. 

### 2.1. Population and Sampling 

The population of this study comprised all nursing students enrolled in HEIs for master’s and doctoral degrees (N = 46). Notably, non-probability, purposive sampling was used to select 23 registered postgraduate nursing students [25,26]. Notably, 16 master’s and 9 doctoral postgraduate nursing students participated in unstructured one-on-one interviews, and data saturation determined the sample size of this study [27]. 

### 2.2. Study Setting 

The study was conducted at the two HEIs in the Limpopo province of South Africa. These two higher education institutions are categorised as historically disadvantaged HEIs, and their setting is rural, located in Capricorn and Vhembe Districts, respectively. The fifth-largest province, Limpopo (one of the nine in the country), covers 10.3% of South Africa’s total geographical area, and approximately 80% of Limpopo’s people live in rural areas [28]. 

### 2.3. Data Collection 

Data were collected through an unstructured one-on-one interview method. One central question was posed to the participants: “Could you kindly explain your experiences of conducting research as an open and distance learning journey at your university?” Researchers conducted interviews in English at venues convenient to participants, and the statements were transcribed verbatim. Interviews lasted for 35–45 min, and data were saturated in 23 postgraduate nursing students. Probing was conducted to obtain more information. Field notes and a digital audiotape were used to capture all interviews conducted in a quiet classroom, free from noise and disturbance.

### 2.4. Data Analysis

The narrative data from interviews were analysed qualitatively through Tesch’s open coding method described by Creswell [29]. The method included the following steps: all transcripts were read carefully to obtain a sense of the whole, a list was made of all similar topics, field notes were coded and categorised, and data were then grouped according to themes and sub-themes. Three themes emerged from the study findings: the importance of conducting research, strategies utilised to succeed with the research project, and experiences associated with conducting research.

### 2.5. Ethical Considerations 

Health Sciences Research Ethics Committee clearance at UL (Ref no: TREC/185/2016) was obtained, and institutional approval was sought from UNIVEN before this study commenced. Importantly, the gatekeeper permission was also obtained from the nursing department of each university before the data collection process. Postgraduate nursing students voluntarily signed the informed consent form before participating in the interview sessions. The protection of information obtained from the participants was in line with the Protection of *Personal Information Act* (POPIA) No. 04 of 2013. Lastly, anonymity and confidentiality were ensured by assigning pseudonyms to all postgraduate nursing students participating in this study. Participants were only identified as Participants 1, 2, or 3 during data collection. 

### 2.6. Measures to Ensure Trustworthiness 

In this study, Lincoln and Guba’s criteria of credibility, transferability, dependability and confirmability were followed to ensure trustworthiness, as cited by Korstjens and Moser [30]. Therefore, credibility was ensured through a dense description of the collected data, during which the researcher interviewed participants and prolonged engagement; data collection was completed over a month. Confirmability in this study was ensured by writing filed notes and using digital audiotapes during unstructured one-on-one interviews. Transferability was enhanced by using the purposive sampling method to select postgraduate nursing students to participate in the study. Dependability was achieved by the third author of the study, who independently coded the results. 

## 3. Results and Discussion 

### Demographic Data of Postgraduate Nursing Students 

Twenty-three (23) postgraduate nursing students, aged between 19 and 59 years, were interviewed for this study. Of these, 61% were female, and 39% were male. Furthermore, 70% of participants were single, while 30% were married, and employment status revealed that 96% of postgraduate nursing students were full-time employed, with 04% (01) being unemployed. Subsequently, 61% of the participants were registered with the University of Limpopo, while 39% were registered with the University of Venda. In addition, 61% of all postgraduate nursing students were in the first and second year of their studies, 30% were in the third and fourth year of their studies, and lastly, 9% (02) were in the fifth year of their studies. 

Table 1 summarises the demographic data of postgraduate nursing students who participated in the study. The themes and sub-themes that emerged from their studies are delineated in Table 2. 

Table 2 provides a summary of themes and sub-themes of the postgraduate nursing students’ experiences of conducting research as an open and distance learning journey. 

Three themes and seven sub-themes emerged from the study, as indicated in Table 2 above, and are discussed lengthily as follows.

**Theme 1: The reasons behind engaging in postgraduate studies**

It emerged that postgraduate nursing students understand the reasons behind engaging in postgraduate studies, which include (*i*) the provision of evidence-based solutions and (*ii*) personal, career, and academic development.

*Sub-theme 1.1: Provision of evidence-based solutions*

Participants believe that nursing research provides solutions to the healthcare problems they experience in their workplace and keeps them abreast of developmental changes in the healthcare sector. The participants share a strong sense of valuing the contribution of research. This was confirmed by study participants who said:

“*It has taught me that when treating a patient, you don’t consider subjective and objective data only, there is more to a symptom*”.

(Participant 12)

“*There are so many problems we are encountering at the hospitals, which I believe can only be solved through research*”.

(Participant 08)

“*Research is important because it helps us nurses to address health problems scientifically, even though it is tough studying while working*”.

(Participant 23)

The impact of evidence-based practice echoes across nursing practice, education, and science. The above assertions are supported by Brooke, Hvalič-Touzery, and Skela-Savič, who point out the importance of evidence-based practice and research in exploring and understanding poor healthcare to improve patient care and safety [31]. Nursing research must be conducted in addition to clinical practice because the results lay a solid foundation for patient care and practice improvement. Laske and Kurz argue that students should be capacitated with a robust research foundation during undergraduate teaching and learning to address clinical competencies [32]. Importantly, research provides evidence-based knowledge and solutions to assess, evaluate, and deliver effective nursing care. Similarly, Al Qadire asserts that nurses have a crucial role in creating and using research evidence, which calls for understanding the fundamentals of evidence-based practice [33]. The author further argues that using research findings to support clinical practice could improve patient clinical outcomes, lower healthcare costs, and raise the standard of treatment. Notably, the basis for evidence-based nursing practice is research. One participant said the following:

“*With research, you have all answers, and research helps develop health in general and helps on how to manage different conditions*”.

(Participant 22)

Brooke, Hvalič-Touzery, and Skela-Savič report that student nurses agree that research ensures the effective implementation of evidence-based practice, providing them with confidence, knowledge and empowerment in their clinical practice [31]. The call for evidence-based quality improvement and healthcare transformation underscores the need to redesign effective, safe, and efficient care. Such initiatives should include education, curricula, realignment, theory development, and the development of a national research network to improve engagement in scientific research. These results are in contrast to those of a study conducted by Dagne and Beshah in public hospitals in the Amhara Region of Ethiopia, which found that nurses and midwives frequently used guidelines, hospital protocol, and training manuals when making clinical decisions and very infrequently used research in clinical practice [34]. These results concur with the findings of surveys among Belgian nurses conducted by Lanssens, Goemaes, Vrielinck, and Tency [34]. Results showed that nursing practitioners rarely use research and scientific literature and rely on their colleagues’ practical knowledge and guidance from physicians. Evidence-based practice is an approach that requires decisions about health care to be based on the best available, current, and valid evidence. For these reasons, nurses are becoming knowledgeable that research can effectively broaden and inform healthcare decisions.

*Sub-theme 1.2: personal, career, and academic development* 

The findings of this study have revealed that nurses conduct research for personal, career, and academic development. This is evident when participants in this study said the following:

“*I love research, and I have joined master’s programme because I want to advance myself and become a professor one day*”.

(Participant 18)

“*I do research because I am motivated by one professor who is a research professor. I wish to be like her and be able to be recognised for better positions at work*”.

(Participant 12)

“*It is important as a nurse to research because we do statistics at work, and we need to be able to interpret, make sense of them, and improve our services. I want to be that nurse who is a research expert in my ward*”.

(Participant 04)

Although a salary increase and promotion are the most motivating factors for employees in the workplace to pursue postgraduate education, the findings of the study conducted by Nashwan and colleagues reveal that the pursuit of postgraduate qualifications among nurses is an intrinsic desire to improve the quality of health care rendered and also for personal and professional development [35]. Postgraduate qualifications improve the professional nurse’s educational, research, and administrative duties, according to Mbombi and Mothiba [4]. These authors further argued that having more clinically trained nurses with postgraduate qualifications offers a chance to preserve the nursing fraternity and enhance healthcare delivery.

**Theme 2: Factors impacting postgraduate studies’ success** 

The results of this study indicated that for postgraduate nursing students to successfully complete their postgraduate research projects, they must devise winning tactics. Two sub-themes emerged: (i) positive attitude versus negative attitude towards conducting research outlined, including (ii) an outline of the importance of technology in conducting a research project.

*Sub-theme 2.1: The impact of participants’ attitude towards research* 

Participants revealed positive and negative attitudes towards conducting postgraduate research. Disinterest in postgraduate research education is a barrier to progressing and completing the research dissertation and theses, as postgraduate students fail to commit and advance. This was corroborated by the following research participant, who stated the following:

“*…Research is a really good thing; you are able to learn more and understand people better*”.

(Participant 20)

The finding of this study reveals that postgraduate nursing students had a positive experience with research in nursing, which is in line with recent studies by Abun, including that of Ross and Burrell, which reveals that most of the participants had a positive attitude towards nursing research [36,37]. Nurses’ positive experiences towards research are discordant with their involvement in research activities. Hence, Loura et al. recommend the involvement of nurses in various research projects to elicit the integration of the knowledge they have acquired into practice and to promote evidence-based practices [38]. On the contrary, the participants of this study alluded that conducting nursing research is a stressful, complicated, challenging, and complex exercise. Participants spelt out the following:

“*Research is very difficult, and I don’t see myself conducting it again in future because of its ups and downs and knowledge required*”.

(Participant 06)

“*Research is interesting; however, it is difficult to do research while working. As such, I am unable to keep up with the time and to show my full potential in the programme. However, because I want to be a researcher, I keep pushing and I am not going to quit*”

(Participant 14)

Similar findings were found in the study by Amoo and Gbadamosi, where only 37% of participants said they enjoyed conducting research since many of them thought it was stressful, challenging, and about a complex subject [39]. There is a need to reinforce postgraduate nursing research education as it can positively affect nurses’ attitudes toward nursing research, resulting in better patient treatment and care. Based on the findings of the study conducted by Einarsen and Giske, research participants felt that their participation in qualitative research in a hospital setting had increased their interest in nursing research [40]. Subsequently, many participants agreed that their involvement has equally increased their understanding of the research process and insight into the research methods. Nurses’ positive attitudes towards research are discordant with their involvement in research activities.

*Sub-theme 2.2: Good versus poor knowledge and skills related to the use of technology in postgraduate studies* 

The study’s findings reveal that postgraduate students who are conducting individual postgraduate research need information about technology to use their computers, especially at home. Participants maintained the following:

“*…We are previous university students, and we never experienced a problem because we were introduced to subjects by librarians who oriented us about IT and how to conduct a literature search, and it was emphasised that we need to be computer literate as we started with research projects*”.

(Participant 03)

In support of the findings of this study, Marković emphasises that to recognise, gather, process, and apply scientific data in nursing practice and research, technology literacy must be integrated and prioritised in postgraduate research education [41]. Conversely, the study conducted by Yende highlights that it is vital for higher education institutions to establish language skills, communication skills, and computer literacy, as they play an essential role in enhancing postgraduate students’ knowledge and positive experience in research [41]. Advanced technology in higher education institutions generally improves the quality of teaching and learning and elicits research interest among students. Equally, postgraduate nursing students indicated that they experienced challenges concerning scientific and academic writing since English was not their home language, which impacted the advancement of their postgraduate research dissertations and theses. One participant avers the following:

“*…When I started with the programme, I struggled with academic writing which delayed my progress. I think this should be addressed during the workshop we attend at the beginning of the course*”.

(Participant 11)

Singh highlighted that international postgraduate students in Malaysian universities experience challenges and barriers with their academic writing and reading skills because English is not their first language [23]. Since English is not most postgraduate students’ native language and research dissertations and theses are often written in English, academic writing will continue to be difficult for postgraduate students. For this reason, higher education institutions’ postgraduate research education curriculum should include academic writing proficiency. In the study by AlMarwani, the enculturation of novice postgraduate students into academia by obtaining academic writing principles is recommended in higher education institutions [42].

**Theme 3: Description of challenges associated with conducting research** 

This theme emerged when postgraduate students continually conversed about their experiences conducting postgraduate nursing research as an open and distance learning journey. Notably, three themes emerged, namely: *(i)* barriers experienced when conducting research, *(ii)* challenges in obtaining participants’ informed consent for participation, and lastly, *(iii)* variable experiences of mentoring from the research supervisor.

*Sub-theme 3.1: Barriers experienced when conducting research* 

The participants in this study noted obstacles to conducting postgraduate research, including a lack of time because they are employed on a full-time basis and not limited to a lack of training and expertise in conducting research. According to the participants in this study:

“*…time was really a problem because research requires ample time, looking at literature review and also going out there to recruit for participants*”.

(Participant 10)

“*…I have a family to look after and care for, so my time for research is limited*”.

(Participant 16)

This study’s findings corroborate the results of the study conducted by Konwar and Kalita, in which resource-related problems emerged, and 59.61% of participants realised that lack of time was the main barrier to conducting research [43]. Furthermore, participants in this study expressed that the research ethics committee and approval from the Department of Health (DoH) hindered their research progress as they took a long time to review applications. Delayed outcomes on ethics and approval from involved research stakeholders, including lack of research mentoring, negatively impede the postgraduate research progress and throughput rate. Therefore, a reinforced research mentorship programme and feasible turn-around time for ethics and approval to conduct research should be established.

*Sub-theme 3.2: Challenges in obtaining participants’ informed consent for participation* 

The participants expressed the challenge of recruiting participants for their studies, as some of them were reluctant to be involved, citing various reasons. Participants in this study indicated the following:

“*I thought people from our profession understood what research was, unfortunately, people thought when interviewing them, the information is taken elsewhere, and their names will be revealed*”.

(Participant 13)

“*It became a problem when conducting research wherein the participants are colleagues, and they don’t want to participate*”.

(Participant 15)

“*It took me a long time to get participants because the nurses, as my participants’ thought participating in the study, they are helping me to pass, so they expected to be paid*”.

(Participant 19)

The involvement of nurses in a research project is crucial as it links patient care with evidence-based interventions. Coyne et al. allude that workplace culture is among other factors that hinder nurses from participating in the research project, as culture may be supportive or, as commonly found, unsupportive [26]. Some nurses enjoy participating in research, and their organisation values research and encourages staff members to get involved in research projects. Therefore, it is crucial for research to successfully recruit nurses in order to gain insight into current practices and suggest strategies to enhance patient outcomes, policy, and care delivery.

*Sub-theme 3.3: Mixed experiences of mentoring from the research supervisor* 

Research mentor or supervisor support is viewed differently by postgraduate nursing students in this study. Participants stated that they were motivated by their mentors. The following statement from participants bore this out:

“*…I was motivated by clinical nurse practitioners as I received referrals from them; they know what is known by doctors, and they further displayed independence during practice, which encouraged me to work hard to be like them, especially those who have conducted research before*”.

(Participant 02)

“*My supervisor was very supportive and took a mentorship role in taking me through the research journey. She also invited me to workshops and conferences so that I can develop as an emerging researcher*”.

(Participant 09)

However, some participants experienced a lack of adequate academic guidance from research mentors or supervisors. Research supervisors are responsible for mentoring postgraduate students in the research processes and methods. Mentorship is believed to result in improved retention, quality, progress, and completion rates of the research dissertations and thesis [19,44]. From these findings, research supervisors should set up enough time to meet with postgraduate students one-on-one and in cohort sessions. Additionally, regular consolidated time blocks away from the higher education institution in the form of writing retreats can provide research supervisors and postgraduate students with the chance to work productively on their research theses [18]. Constant, thoughtful supervision and availability are critical to successful postgraduate research completion, quality, and throughput rate. Conversely, to supervise postgraduate research effectively, the supervisor must be competent in research processes and methods, reflect on research practices, and provide constructive and timely feedback. 

## 4. Recommendations 

Based on the findings of this study, efforts to encourage postgraduate nursing students to enrol, complete, and enjoy research should be a priority. Higher education institutions (HEIs) should support postgraduate students who are struggling with technology and academic writing through one-on-one and group mentoring and workshops. All of this could be achieved by assisting the students to undertake evidence-based research with practical applications, regular communication with the students, practical orientation to literature search, and quick processing of the ethical research approvals by the ethical review committee. The study recommends further research on the strategies to improve the ODL of postgraduate nursing students at historically disadvantaged HEIs. 

## 5. Limitations to the Study

The study has been conducted among two rural universities in Limpopo. The results thereof can, therefore, not be generalised.

## 6. Conclusions 

The findings indicate that students generally have variable experiences regarding completing research projects as a valuable learning experience through ODL mode. The negative experiences are related to the limited duration of study time complicated by delayed ethical clearance and approval, academic writing, and the challenge of recruiting participants. Research is vital as it leads to evidence-based practice that could be used to solve healthcare problems. The student–supervisor mentorship is perceived to be very important in ensuring that students make consistent progress and successfully present their theses on time. The study will, therefore, assist HEIs offering postgraduate programmes in nursing in realising the challenges faced by students and coming up with strategies to combat the challenges. When these challenges are addressed, the institution’s graduation throughput will increase, including student satisfaction. 

## Figures and Tables

**Table 1:** Demographic data of postgraduate nursing students.

*Demographic Variables*	*Population, n (%)*
Gender	
Female	14 (61%)
Male	09 (39%)
Age	
19–29	05 (22%)
30–39	08 (35%)
40–49	07 (30%)
50–59	03 (13%)
Marital Status	
Single	16 (70%)
Married	07 (30%)
Employment Status	
Employed	22 (96%)
Unemployed	01 (4%)
Institution enrolled with	
University of Limpopo	14 (61%)
University of Venda	09 (39%)
Qualification enrolled for	
Master’s Degree	16 (70%)
Doctoral Degree	07 (30%)
Years of study	
1st–2nd year	14 (61%)
3rd–4th year	07 (30%)
>5th	02 (09%)
*Total*	*23*

**Table 2:** Themes and Sub-themes.

*Themes*	*Sub-Themes*
The reasons behind engaging in postgraduate studies	*1.1.* *Provision of evidence-based solutions*
	*1.2.* *Personal, career, and academic development*
2. Factors impacting postgraduate studies’ success	*2.1.* *The impact of student’s attitude towards research*
	*2.2.* *Good versus poor knowledge and skills related to the use of technology in postgraduate studies*
3. Description of challenges associated with conducting research	*3.1.* *Barriers experienced when conducting research*
	*3.2.* *Challenges in obtaining participants’ informed consent for participation*
	*3.3.* *Mixed experiences of mentoring from the research supervisor*

## Data Availability

The authors cannot share the study data due to the institution’s ethical constraints.

## Public Involvement Statement

No public involvement in any aspect of this research.

## Guidelines and Standards Statement

This manuscript was drafted against the Consolidated criteria for reporting qualitative research. A complete list of reporting guidelines can be accessed via the equator network: https://www.equator-network.org/ (accessed on 16 January 2024).
